# Hemodilution is associated with underestimation of serum creatinine in cardiac surgery patients: a retrospective analysis

**DOI:** 10.1186/s12872-021-01879-w

**Published:** 2021-01-31

**Authors:** Jifu Jin, Jiarui Xu, Sujuan Xu, Jiachang Hu, Wuhua Jiang, Bo Shen, Chunsheng Wang, Jie Teng, Xiaoqiang Ding

**Affiliations:** 1 Department of Nephrology, Zhongshan Hospital, Shanghai Medical College, Fudan University, 180 Fenglin Road, Shanghai, 200032 China; 2 Shanghai Medical Center of Kidney Disease, Shanghai, China; 3 Shanghai Institute of Kidney and Dialysis, Shanghai, China; 4 Shanghai Key Laboratory of Kidney and Blood Purification, Shanghai, China; 5 Department of Cardiology, Shanghai East Hospital, Tongji University, Shanghai, China; 6 Department of Cardiac Surgery, Zhongshan Hospital, Shanghai Medical College, Fudan University, Shanghai, China

**Keywords:** Acute kidney injury, Fluid balance, Cardiac surgery, Diagnosis, Prognosis

## Abstract

**Background:**

Fluid overload is related to the development and prognosis of cardiac surgery-associated acute kidney injury (CSA-AKI). The study is to investigate the influence of serum creatinine (SCr) corrected by fluid balance on the prognosis of patients with cardiac surgery.

**Methods:**

A retrospective study was conducted in 1334 patients who underwent elective cardiac surgery from January 1 to December 31, 2015. Kidney Disease: Improving Global Outcomes (KDIGO) criteria for AKI were applied to identify CSA-AKI. SCr was measured every 24 h during ICU period and was accordingly adjusted for cumulative fluid balance. Changes in SCr, defined as ∆Crea, were determined by difference between before and after adjustment for cumulative fluid balance. All patients were then divided into three groups: underestimation group (∆Crea ≥ P_75_), normal group (P_25_ < ∆Crea < P_75_) and overestimation group (∆Crea ≤ P_25_).

**Results:**

The incidence of AKI increased from 29.5% to 31.8% after adjustment for fluid balance. Patients in underestimation group showed prolonged length of ICU stay compared with normal group and overestimation group (3.2 [1.0–4.0] vs 2.1 [1.0–3.0] d, *P* < 0.001; 3.2  [1.0–4.0] vs 2.3 [1.0–3.0] d, *P* < 0.001). Length of hospital stay and mechanical ventilation dependent days in underestimation group were significantly longer than normal group (*P* < 0.001). Multivariate analysis showed age, baseline SCr and left ventricular ejection fraction were independently associated with underestimation of creatinine.

**Conclusions:**

Cumulative fluid balance after cardiac surgery disturbs accurate measurement of serum creatinine. Patients with underestimation of SCr were associated with poor prognosis.

## Background

Acute renal failure (ARF) develops in approximately 2% of patients after cardiac surgery and is associated with an excessive mortality rate up to 60%-80% [1–4]. Unfortunately, ARF is not recognized as a definition for the disease status ranging from quantitative and qualitative alterations [5]. Meanwhile, subsequent studies confirmed that small changes in serum creatinine were associated with an increased mortality [6]. The new term acute kidney injury (AKI) reflecting the complex continuum of renal dysfunction was gradually accepted. As the first-line treatment of critically ill patients, fluid resuscitation may cause positive fluid balance during treatment, which frequently results in a relative increase in body weight of 10%-15% in a short time [7, 8]. However, recent studies have illustrated that positive fluid balance was associated with worse outcome in critically ill patients with AKI [9–12]. Stein A et al*.* found both fluid overload and changes in serum creatinine were related to the adverse outcomes, including death, infection, bleeding, arrhythmia and pulmonary edema [13]. Furthermore, positive fluid balance was associated with recognition, staging and outcome of AKI in patients with acute respiratory distress syndrome or undergoing cardiac surgery [14–17]. Macedo et al*.* found that fluid accumulation may induce underestimation of the severity of AKI and increase the time to identify a 50% relative increase in serum creatinine [18]. Based on these results, the aim of our study is to investigate the influence of discrepancy of serum creatinine on the prognosis of patients with cardiac surgery, and moreover, to explore underlying risk factors for underestimation of serum creatinine.

## Methods

### Patients

Patients who underwent cardiac surgery between January 1 and December 31, 2015 at the Department of Cardiovascular Surgery, Zhongshan Hospital, Fudan University were consecutively included in our study. Inclusion criteria were adult patients (< 75 years) who received elective cardiac surgery with or without cardiopulmonary bypass (CPB), including coronary artery bypass graft (CABG) and valvular surgery. Exclusion criteria were patients who had preexisting renal dysfunction requiring renal replacement therapy or had a baseline creatinine ≥ 4 mg/dl; patients who died within 24 h after surgery as well as patients who received cardiac transplantation or aortic aneurysm surgery. The Institutional Ethics Committee of the Zhongshan Hospital (B2018-175) granted permission for data collection and informed consent was waived due to the retrospective design of the study.

### Data collection

Acute Physiology and Chronic Health Evaluation II (APACHE II) score was used to assess severity of illness at intensive care unit (ICU) admission [19]. Intraoperative parameters, such as CPB and cross-clamp duration, types of surgery and ultrafiltration during extracorporeal circulation were recorded as well. Fluid input and output were obtained at least 24 h since ICU admission or until discharge from the ICU, whichever occurred first. Insensitive loss of fluid was not taken into account in our study.

### Definitions

Kidney Disease: Improving Global Outcomes (KDIGO) definition was utilized to identify cardiac surgery-associated acute kidney injury (CSA-AKI) [20]. The dilutional effect of fluid overload on the diagnosis and staging of CSA-AKI was assessed.

Cumulative fluid balance was calculated based on total fluid input and output in every 24 h. Patients’ admission weights were utilized to estimate baseline total body water (TBW). TBW = 60% of baseline weight in kilogram at admission [18].

Baseline serum creatinine was obtained immediately prior to surgery. Maximum serum creatinine was defined as the highest value within 48 h after cardiac surgery. During the postoperative ICU period, serum creatinine was measured at least once every 24 h. The adjusted serum creatinine was calculated with the formula used in the previous studies [14, 18, 21]. Adjusted creatinine = serum creatinine × [1 + (cumulative fluid balance in L/admission weight in kg × 0.6)]. The difference of adjusted and unadjusted serum creatinine was defined as ∆Crea.

### Groups

**Fig. 1:**
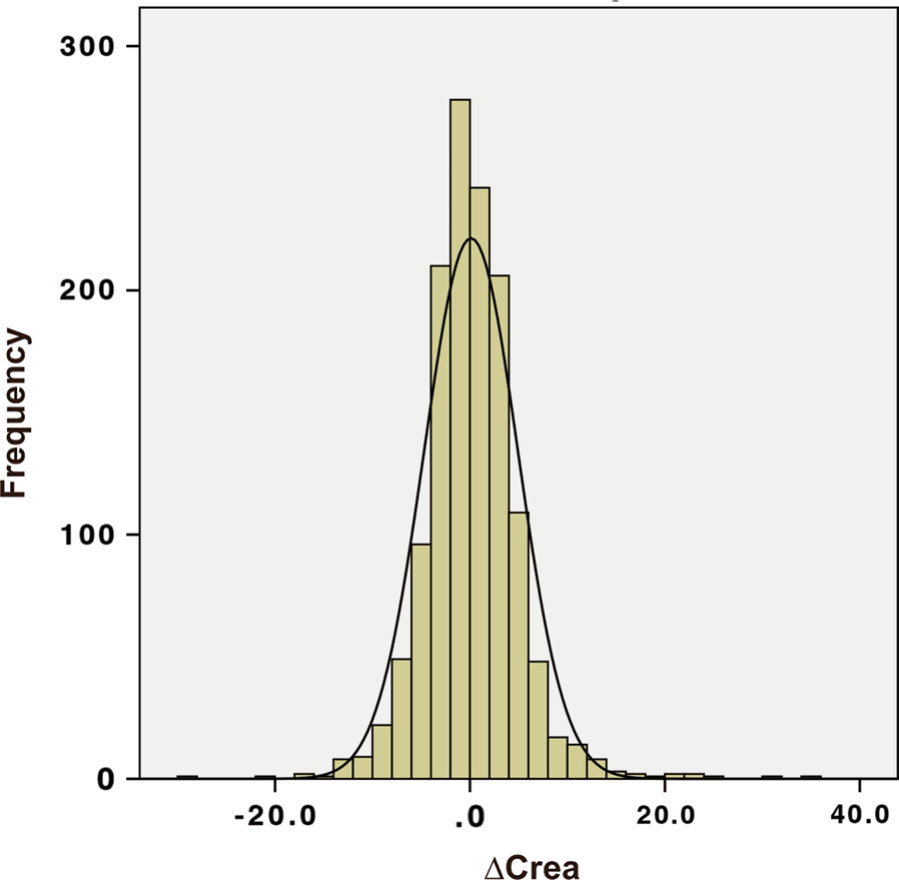
The normal distribution of ∆Crea. Mean of ∆Crea = 0.11; Standard deviation of ∆Crea = 4.81; N = 1334

Given that the normal distribution of ∆Crea (Fig. 1), patients were divided into three groups: underestimation group (∆Crea ≥ P_75_), normal group (P_25_ < ∆Crea < P_75_) and overestimation group (∆Crea ≤ P_25_). P_25_ and P_75_ are used to indicate the 25_th_ and the 75_th_ percentile value of ∆Crea, respectively. The primary outcome is in-hospital mortality rate and its relationship to the ∆Crea. The secondary outcome is length of ICU stay, total length of hospital stay as well as mechanical ventilation dependent days across these groups.

### Statistical analysis

Continuous variables were expressed as means (standard deviations) or medians (interquartile range, IQR). Comparisons across three groups were made using analysis of variance (ANOVA) for normally distributed variables and Kruskal–Wallis test for non-normally distributed variables. Categorical variables were expressed as counts with proportions and were compared using chi-square test or Fisher’s exact test where appropriate. Differences in recognition of AKI before and after adjustment for cumulative fluid balance were evaluated with McNemar’s test. Consistency of AKI diagnosis and staging was assessed in Cohen’s weighted kappa coefficient. Univariate analysis was performed regarding underestimation of serum creatinine as an outcome variable. Risk factors of significance in univariate analysis were further included in multivariate analysis to confirm independent risk factors for underestimation of serum creatinine. A two-sided *P* value of < 0.05 was considered to be statistically significant. All analyses were performed using SPSS 11.0 software (ver. 18.0, SPSS Inc., US).

## Results

### Basic characteristics

A total of 1334 patients (776 men) with a mean age of 56 years were investigated. Among these patients, 20.8% (278/1334) cases received off-pump CABG whereas 79.2% (1056/1334) cases received on-pump cardiac surgery including valvular surgery and CABG plus valvular surgery. All patients were divided into three groups based on ∆Crea level (Table 1). More patients had diabetes mellitus, hypertension, previous contrast exposure, surgery without CPB in underestimation group. Accordingly, patients in underestimation group received more crystalloid and colloid fluids during perioperative period. Man sex, weight, and history of chronic kidney disease, acute coronary syndrome as well as stroke were similar across ∆Crea groups. Differences were noted across groups with respect to the age, left ventricular ejection fraction, baseline creatinine, APACHE II score, CPB and cross-clamp duration.

**Table 1:** Basic characteristics of the patients according to the differences between adjusted and unadjusted postoperative serum creatinine

	Underestimation n = 353	Normal n = 641	Overestimation n = 340	*P* value
Demographic data				
Male [n (%)]	228 (64.6)	338 (52.7)	210 (61.8)	0.687
Age (years)	61 ± 11	56 ± 12	52 ± 13	< 0.001
BMI (kg/m^2^)	23.2 ± 3.0	23.3 ± 3.3	22.9 ± 3.4	0.314
Comorbid conditions [n (%)]				
Hypertension	163 (46.2)	218 (34.0)	97 (28.5)	< 0.001
DM	73 (20.7)	66 (10.3)	20 (5.9)	< 0.001
CKD	6 (1.7)	4 (0.6)	4 (1.2)	0.272
Cancer	3 (0.8)	10 (1.6)	7 (2.1)	0.418
ACS	8 (2.3)	13 (2.0)	1 (0.3)	0.073
Stroke	8 (2.3)	19 (3.0)	6 (1.8)	0.494
Atrial fibrillation	12 (3.4)	25 (3.9)	13 (3.8)	0.921
Contrast exposure [n (%)]	210 (59.5)	356 (55.5)	168 (49.4)	0.027
NYHA III-IV [n (%)]	211 (59.8)	470 (73.3)	266 (78.2)	< 0.001
LVEF (%)	59.7 ± 9.6	61.9 ± 8.6	60.6 ± 9.3	0.006
Preoperative SCr (μmol/L)	87.4 ± 31.8	76.7 ± 17.1	81.9 ± 24.1	< 0.001
APACHE II at ICU admission	13.1 ± 3.4	12.0 ± 3.5	12.7 ± 3.9	< 0.001
Type of surgery [n (%)]				
Off-pump	154 (43.6)	119 (18.6)	5 (1.8)	< 0.001
CABG	154 (43.6)	119 (18.6)	5 (1.8)	< 0.001
On-pump	199 (56.4)	522 (81.4)	335 (98.5)	< 0.001
Valvular surgery	174 (49.3)	424 (66.1)	274 (80.6)	< 0.001
CABG + valvular surgery	25 (7.1)	98 (15.3)	61 (17.9)	< 0.001
On pump-surgery variables				
CPB time (min)	105.3 ± 45.2	91.2 ± 32.3	104.4 ± 72.2	< 0.001
Cross-clamp (min)	63.9 ± 28.6	56.1 ± 30.6	60.1 ± 26.8	0.006
Ultrafiltration (ml)	1311 ± 1417	1727 ± 1036	1891 ± 1268	< 0.001

The data in the table are expressed as mean ± standard deviation or number (%). *P* value is for the comparison among groups

### Incidence of CSA-AKI before and after adjustment

The incidence of AKI based on the KDIGO criteria was 29.5% (with 24.4%, 3.0% and 2.1% in AKI stage 1–3, respectively). The in-hospital mortality rate was 1.05% (14/1334) and incidence of requirement for continuous renal replacement therapy (CRRT) during 48 h after ICU admission was 0.37% (5/1334). After adjustment for cumulative fluid balance, the incidence of AKI elevated to 31.8% (increased from 24.4 to 26.5% in stage 1, decreased from 3.0 to 2.8% in stage 2 and increased from 2.1 to 2.5% in stage 3 respectively). AKI stage only increased by one stage in three groups after adjustment for cumulative fluid balance. An increase was found in only 3.7% of those originally in stage 0, 0.4% in those originally in stage 1 and 0.5% in those originally in stage 2. The percentage of agreement for AKI diagnosis was 94.9% with a kappa of 0.86 (95% confidence interval [CI] 0.83–0.89), whereas percentage of agreement for AKI staging was 93.8% with a kappa of 0.86 (95% CI, 0.83–0.89) after adjustment for cumulative fluid balance (Table 2).

**Table 2:** Diagnosis and staging of AKI before and after adjustment for fluid balance in patients after cardiac surgery

Unadjusted AKI stage	Adjusted AKI stage				Total, N (%)
	0	1	2	3	
0	890 (66.7)	50 (3.7)	0 (0)	0 (0)	940 (70.5)
1	19 (1.4)	301 (22.6)	6 (0.4)	0 (0)	326 (24.4)
2	0 (0)	2 (0.1)	31 (2.3)	7 (0.5)	40 (3.0)
3	0 (0)	0 (0)	1 (0.07)	27 (2.0)	28 (2.1)
Total, N (%)	909 (68.1)	353 (26.5)	38 (2.8)	34 (2.5)	1334

Kappa = 0.86 (95% CI 0.83–0.89) and percentage agreement = 94.9% for AKI diagnosis

Kappa = 0.86 (95% CI 0.83–0.89) and percentage agreement = 93.8% for AKI staging

### Changes in serum creatinine (∆Crea) and outcomes

Patients in underestimation group showed prolonged length of ICU stay compared to the normal group and overestimation group (3.2 [1.0–4.0] vs 2.1 [1.0–3.0] d, *P* < 0.001; 3.2 [1.0–4.0] vs 2.0 [1.0–3.0] d, *P* < 0.001, respectively). Both length of hospital stays and ventilation dependent days in underestimation group were significantly higher than normal group (13.8 [10.0–15.0] vs 12.0 [10.0–14.0] d, *P* < 0.05; 1.7 [1.0–2.0] vs 1.2 [0.5–1.5] d, *P* < 0.001, respectively) (Table 3). There were no statistical differences in the in-hospital mortality rate and the incidence of CRRT across these groups.

**Table 3:** Outcomes of the patients according to the difference between adjusted and unadjusted postoperative serum creatinine

	Underestimation n = 353	Normal n = 641	Overestimation n = 340	*P* value
Outcome variables				
Ventilation dependent days (days)	1.7 [1.0–2.0]	1.2 [0.5–1.5]	1.0 [0.5–1.5]	< 0.001
ICU stay (days)	3.2 [1.0–4.0]	2.1 [1.0–3.0]	2.0 [1.0–3.0]	< 0.001
Hospital stay (days)	13.8 [10–15]	12.0 [10–14]	12.0 [10–15]	0.012
CRRT [n (%)]	0 (0.0)	2 (0.3)	3 (0.9)	0.591
In-hospital mortality [n (%)]	4 (1.1)	5 (0.8)	5 (1.5)	0.154

The data in the table are expressed as median ± interquartile range or number (%). *P* value is for the comparison among groups

### Risk factors associated with underestimation of serum creatinine

Multivariate analysis indicated that factors independently associated with underestimation of serum creatinine due to the cumulative fluid balance were older age (*P* = 0.035), higher baseline serum creatinine (*P* < 0.001), lower left ventricular ejection fraction (*P* = 0.001), and extent of cumulative fluid balance (*P* < 0.001) during ICU stay after cardiac surgery (Table 4).

**Table 4:** Univariate and multivariate analysis with underestimation of serum creatinine as the outcome variable

Variable	Univariate analysis		Multivariate analysis	
	HR (95% CI)	*P* value	HR (95% CI)	*P* value
Sex, Male	1.44 (1.12–1.85)	0.004		
BMI (per 1 kg/m^2^ increase)	1.01 (0.96–1.06)	0.723		
Age (per 1 unit increase)	1.05 (1.04–1.60)	< 0.001	1.05 (1.01–1.10)	0.035
Hypertension (present)	1.81 (1.41–2.33)	< 0.001		
Diabetes (present)	2.71 (1.93–3.81)	< 0.001		
CKD (present)	2.10 (0.72–6.10)	0.172		
NYHA III-IV (present)	2.02 (1.56–2.61)	< 0.001		
Preoperative SCr (per 1 μmol/L increase)	1.01 (1.01–1.02)	< 0.001	1.05 (1.03–1.08)	0.000
LVEF (per 1 unit decrease)	1.02 (1.01–1.04)	0.010	1.10 (1.04–1.17)	0.001
Off-pump surgery (present)	5.35 (4.03–7.09)	< 0.001		
CPB (per 1 min increase)	1.00 (1.00–1.01)	0.061		
Aortic cross-clamp (per 1 min increase)	1.01 (1.00–1.01)	0.014		
Oliguria (present)	1.87 (0.66–5.29)	0.239		
Cumulative fluid balance (per 1 L increase	1.00 (1.00–1.01)	< 0.001	1.007 (1.005–1.009)	0.000

## Discussion

In this retrospective cohort study, we found that after adjusting serum creatinine for the cumulative fluid balance, more patients met KDIGO criteria for CSA-AKI. Patients in underestimation group had worse outcomes than that in normal group or overestimation group in terms of the length of ICU stay, total length of hospital stay and mechanical ventilation dependent days, but not in the incidence of CRRT or in-hospital mortality rate.

Since minimal increase of serum creatinine was associated with adverse outcomes in patients within the ICU setting, precise recognition and accurate assessment of AKI may contribute to the prevention and early intervention of reversible risk factors for AKI [22–24]. Serum creatinine may normally be influenced by several factors, including renal creatinine clearance or creatinine formation or both [25]. Importantly, serum creatinine level can also be affected by dilution effect of fluid resuscitation, which frequently occurs in critically ill patients [6, 26]. Our results indicate that cumulative fluid balance in patients with cardiac surgery underestimates the diagnosis and staging of AKI, which is in accordance with the results from previous studies [14, 18].

Post hoc analysis of Fluids and Catheters Treatment study illustrated that incidence of AKI with acute respiratory distress syndrome was greater in patients managed with liberal fluid protocol than that in conservative fluid protocol after adjustment for fluid balance [14]. Moreover, mortality rate of these patients was similar to those diagnosed with AKI before and after adjustment for fluid balance. Macedo et al. conducted an analysis in patients underwent nephrology consultation for AKI in ICU settings, which showed dilution effect of fluid overload on serum creatinine may delay the diagnosis time for AKI [18]. Previous study focusing on cardiac surgery patients also demonstrated that patients with AKI only after adjustment for fluid balance had intermediate outcomes between non-AKI and classical AKI patients [15]. Similarly, our study demonstrated that patients with underestimation of serum creatinine had prolonged mechanical ventilation dependent days, longer length of ICU stay and hospital stay.

Multivariate analysis of our study also found that after adjustment for relevant risk factors, patients with older age, lower left ventricular ejection fraction, higher baseline serum creatinine and cumulative fluid balance after cardiac surgery were independently associated with the underestimation of serum creatinine. Thus, to minimize underestimation of serum creatinine and improve subsequent predictive ability of poor outcomes, risk factors including age, baseline cardiac function as well as baseline kidney function should be taken into account before fluid administration during perioperative period in cardiac surgery patients.

Nevertheless, there are several limitations in our study. First, as a single center study, regardless of the large cohort of patients, inherent bias of study design still remains to be concerned. Second, the cause of fluid administration was not easily distinguished from our database. Excess fluid administration may be in an effort to improve low cardiac output, and fluid accumulation may be secondary to inflammatory response. Meanwhile, poor outcomes in underestimation group may in part due to the greater colloids infusion via the damage to endothelial glycocalyx [27, 28]. Last, insensitive fluid loss during study period was not calculated, which may influence the accurate measurement of fluid balance, especially within the patients who were intubated during ICU period.

Regardless of these limitations, our study highlights the dilution effect of cumulative fluid balance on the accurate measurement of serum creatinine and further illustrates associated outcomes in cardiac surgery patients, which may benefit physicians to recognize mild AKI via adjustment for cumulative fluid balance. Strikingly, our study identified for the first time that risk factors including age, baseline cardiac function, and preoperative kidney function were independently associated with the underestimation of serum creatinine, which would be beneficial for screening patients at high risk for misinterpretation of postoperative serum creatinine. However, much more advanced studies should be designed to clarify the underlying association between concealed mild AKI and actual changes of renal function, using the combination of serum creatinine and kidney injury biomarkers to timely detect the deterioration of kidney function after cardiac surgery.

## Conclusions

Cumulative fluid overload in patients after cardiac surgery is very common and leads to the underestimation of postoperative AKI. Patients with underestimation of serum creatinine are associated with a substantial detrimental prognosis.

## Data Availability

The datasets used and/or analyzed during the current study are available from the corresponding author on reasonable request.

## Abbreviations

ACS: Acute coronary syndrome
AKI: Acute kidney injury
ANOVA: Analysis of variance
APACHE II: Acute Physiology and Chronic Health Evaluation II
BMI: Body mass index
CI: Confidence interval
CKD: Chronic kidney disease
CPB: Cardiopulmonary bypass
CRRT: Continuous renal replacement therapy
CSA-AKI: Cardiac surgery-associated acute kidney injury
DM: Diabetes mellitus
GFR: Glomerular filtration rate
ICU: Intensive care unit
KDIGO: Kidney Disease Improving Global Outcomes
LOS: Length of stay
LVEF: Left ventricular ejection fraction
NYHA: New York Heart Association
SCr: Serum creatinine
TBW: Total body water
VDD: Ventilation dependent days
